# Mathematical Model and Calibration Procedure of a PSD Sensor Used in Local Positioning Systems

**DOI:** 10.3390/s16091484

**Published:** 2016-09-15

**Authors:** David Rodríguez-Navarro, José Luis Lázaro-Galilea, Ignacio Bravo-Muñoz, Alfredo Gardel-Vicente, Francisco Domingo-Perez, Georgios Tsirigotis

**Affiliations:** 1 Department of electronics, University of Alcalá, Alcalá de Henares, Madrid 28801, Spain; Lazaro@depeca.uah.es (J.L.L.-G.); ibravo@depeca.uah.es (I.B.-M.); alfredo@depeca.uah.es (A.G.-V.); fco.domingo@depeca.uah.es (F.D.-P.); 2 Informatics Engineering Department, Eastern Macedonia and Thrace Institute of Technology, Kvala 65404, Greece; tsirigo@teikav.edu.gr

**Keywords:** positioning, measurement, PSD, LPS, calibration, infrared

## Abstract

Here, we propose a mathematical model and a calibration procedure for a PSD (position sensitive device) sensor equipped with an optical system, to enable accurate measurement of the angle of arrival of one or more beams of light emitted by infrared (IR) transmitters located at distances of between 4 and 6 m. To achieve this objective, it was necessary to characterize the intrinsic parameters that model the system and obtain their values. This first approach was based on a pin-hole model, to which system nonlinearities were added, and this was used to model the points obtained with the nA currents provided by the PSD. In addition, we analyzed the main sources of error, including PSD sensor signal noise, gain factor imbalances and PSD sensor distortion. The results indicated that the proposed model and method provided satisfactory calibration and yielded precise parameter values, enabling accurate measurement of the angle of arrival with a low degree of error, as evidenced by the experimental results.

## 1. Introduction

This study is related to the development of an LPS (local positioning system) based on IR signals and PSD sensors, used to obtain the position of mobile agents in indoor environments. A variety of technologies is used to implement an LPS, depending on the accuracy, range and cost constraints of each application. Examples of commonly-used technologies include: infrared, ultrasound, video and radio frequency. Our research group is currently developing an LPS based on the emission and reception of near infrared (NIR) signals. As reported in [1], this technology provides positioning accuracies of between 1 cm and 10 cm, with a range of 1 m to 5 m.

Regardless of the technology used for detecting the position of a mobile agent, LPS systems are based on strategies, such as: time of arrival/time of flight (ToA/ToF), which measure the time taken to arrive from signal emission to reception and, therefore, measure the distance between the sender and each receiver; time differential of arrival (TDoA), which measures the difference between times of arrival at different receivers, and thus, three or more receivers are necessary to obtain the position; angle of arrival (AoA), which measures the angles of arrival at each receiver, and therefore, the position of a mobile agent can be obtained with two receivers; and received signal strength (RSS), which extracts the distance between the mobile agent and the receiver based on the strength of the signal reaching each receiver.

ToA and ToF are used in systems based on interferometry [2], time of flight cameras [3,4] or ultrasound [5], in which the sender and receiver share hardware and can detect when the signal is emitted and received, and therefore, measure signal flight time or the phase difference of arrival (PoA-PDoA). The sender and the receiver are distant, so they must be synchronized in order to obtain the propagation times. With high speed signal propagation, as in the case of light or any other electromagnetic signal, synchronization is extremely difficult. However, with TDoA/DoTF (PDoA), the sender and receiver do not need to be synchronized, but the different receivers do, as is the case of the IR-based LPS being developed by our research group [6,7]. In these cases, receivers are synchronized by using a wired signal clock.

Another position determination system is RSS [8,9], a method based on estimating the distance between the sender and the receiver by means of the strength of the signal that reaches the sensors nodes. Position determination can be performed using absolute measurements, for example by estimating the distance between sender and receiver in each node or from differences in the strength of the signal received, performing the same measurements as with TDoA. In the case of infrared, use of ToA/ToF entails the disadvantage that the power received depends on the power transmitted, which is not stable over time and is also sensitive to environmental conditions.

In AoA-based systems, commonly used in digital camera systems [10,11], it is possible to obtain the position using stereoscopic geometry. This strategy is applied to PSD sensors, as reported in [12,13,14]. Once the distances and differences between them, the signal strength and the angles of arrival have been measured, the position of the mobile agent can be determined by trilateration (ToA/ToF, TDoA, PDoA) or RSS and triangulation (AoA).

**Figure 1:**
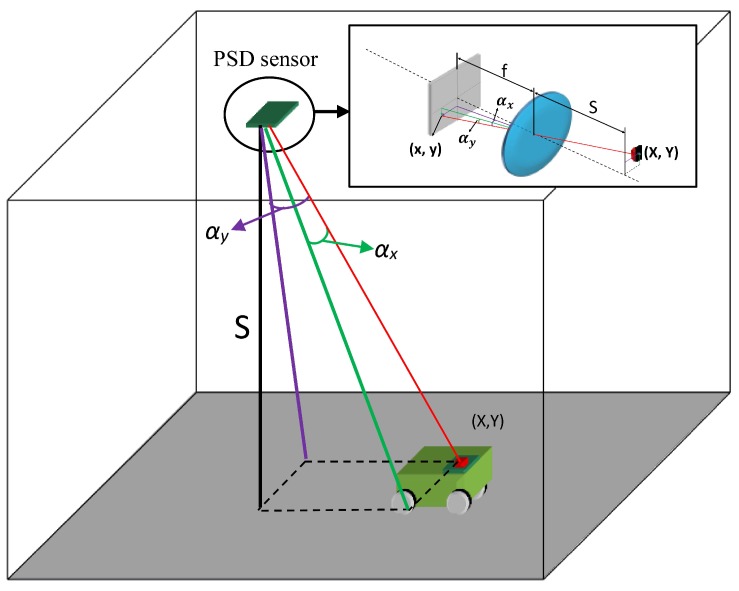
LPS.

Here, we propose a method for measuring the angle of arrival using a PSD sensor, in order to determine the direction of arrival of the optical beam from mobile agents in the environment. To this end, given that an optical system is attached to the PSD with a non-ideal behavior with respect to the theoretical pin-hole model used, it was necessary to perform a geometric calibration to determine the intrinsic parameters of the system, thus enabling us to accurately determine the angle of arrival of the beam. As explained in [15], electrical calibration is required prior to performing a geometric calibration, in order to rectify errors due to imbalances in the amplification channels of the electronic circuit employed to amplify the signals delivered by the PSD, which would hinder the determination of the point of impact (prior to determination of the orientation). The LPS proposed by our group (Figure 1) consists of a sensory system (PSD sensor + optical lens) located in the environment and NIR transmitters carried by the mobile agents.

The 3D position of the NIR transmitter is determined with a single PSD sensor based on the measurements of two parameters. The first of these, the subject of this article, is the angle of arrival, and the second, the determination of which is not discussed here, is the distance between the PSD sensor and the NIR transmitter.

The parameters of interest shown in the Figure 1 are as follows: the point in the environment where the NIR transmitter is located is (X,Y); the impact of its image on the PSD sensor surface is (x,y); the angles of arrival are $\left(\alpha_{x},\alpha_{y}\right)$ for the axes *x* and *y*, respectively; *S* is the distance between the PSD sensor and the plane in which the NIR transmitter moves ; and *f* is the focal length of the optical system attached.

Several systems that use PSD sensors, such as [16], use collimated laser beams, and therefore, the PSD sensor is not attached to an optical system; however, they also require calibration, since the behavior of the PSD presents distortion that must be corrected, as explained in [17]. In [18], laser scanning was used to correct PSD sensor distortion, yielding micron-level accuracy and making it possible to adjust the measurements obtained with the movements performed by the laser. This procedure enabled accurate modeling of PSD sensor distortion, providing a system with very good resolution that was very stable to vibration, as well as very controlled test conditions. Similarly, sweeping with a laser the surface of the PSD sensor in [19], they correct this distortion using neural networks. In other studies, such as [12], stereo measurements have been used to perform calibration. However, this approach is not based on the computation of the intrinsic parameters, but on obtaining the relationship between the environment and the optical system. Since it does not adjust the intrinsic parameter values (optical center, focal length and distortion parameters), the distance measurement will contain an error that can only be corrected by calibrating the real intrinsic parameters.

In our case, due to the few works that there are about calibration methods for PSD sensors with an optical system attached, where the intrinsic parameters are required for the calculation of measures of the angle of incidence, we have chosen calibration methods used for cameras.

The geometric calibration described below is closely related to calibration methods used for cameras, since the mathematical models applied for cameras are generally based on the pin-hole model [20], the same model that we have used for the PSD sensor system. This is a linear model that relates points in the environment to points in the image, depending on the intrinsic linear parameters of the optical system (without distortion, since the model does not have a lens), and the extrinsic linear parameters (rotation, translation). However, despite the similarity, there are differences, because cameras are array detectors with millions of receiver cells (space is discretized), whereas a PSD is an analog sensor that delivers very small currents (pA), with all of the problems and errors that this entails. The parameters corresponding to the effects produced by the lens attached to the PSD sensor, such as radial and tangential distortion, were added to the pin-hole model, as were the corresponding distortion parameters presented by the PSD sensor itself. Once we obtained the proposed mathematical model of the system, it was necessary to develop a method to obtain the various parameters. The several existing methods can be grouped into: (a) those based on three-dimensional templates; and (b) those based on two-dimensional templates. Both types are based on geometric patterns, such as cubes, rods, checks and circles.

Methods using 3D templates include those presented in [21,22,23], which can obtain the camera model parameters from a single image. The disadvantages and key factors are that the calibration template must be accurate, since only one image is used and any error in template production would lead to erroneous calibration. In addition, the template must be correctly positioned so that all sides are visible. In the present case, in which the templates are constructed from IR, the SNR of the signals arriving at the PSD sensor should be as high as possible, and therefore, the calibration template must point towards the sensor, which would not be possible with a 3D template.

Methods based on 2D templates include those presented in [24,25,26]. The advantage of these methods is that they are flexible, since it is not necessary to know the position in which the calibration template has been placed. Thus, in our case, all of the LEDs can point towards the same orientation. Furthermore, these methods also yield the best results. For this study, we used a 2D calibration template manufactured with IR diodes that emit at the wavelength to which the PSD sensor is most sensitive. To model our proposal, we used an adapted version of Zhang’s method [26], which is among the most flexible methods, since it allows more images to be added to the calibration process, thus reducing error in the parameters’ value determination. This is an important feature of the system, because the calibration template has a number short reduced points (LEDs), being very sensitive to errors; thus, the system will be rendered more robust by using more images.

This paper is organized as follows: in Section 2, we explain the mathematical model of the optical system (PSD sensor + lens) based on the pin-hole model; in Section 3, we describe the proposed calibration method used to obtain the optical system parameters, and we analyze mobile robot positioning error according to errors in the intrinsic parameters of the system (sensitivity to errors); in Section 4, we report simulations using the proposed models and methods; in Section 5, we describe experimental PSD sensor calibration tests performed with two different lenses; and in the final section, we present our conclusions.

## 2. System Modeling

**Figure 2:**
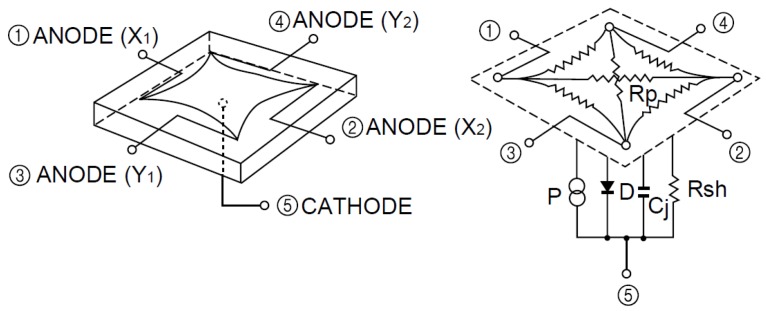
Equivalent circuit of the PSD pin-cushion (image courtesy of Hamamatsu, obtained from the PSD technical information).

The complete optical system consists of a pin-cushion-type PSD sensor attached to a lens. The PSD sensor applied was a photodiode with four anodes and one cathode, as shown in Figure 2.

The point (x,y) at which a beam impacts on the surface of this type of sensor can be calculated using Expressions (1) and (2), developed specifically for this type of PSD sensor, where ($L_{x}$ and $L_{y}$) are the PSD dimensions and $V(x_{i},y_{i})$ are the signals that have been amplified because PSD sensor output currents are very low and considerable error could be generated when digitizing them. The signals must be previously amplified using transimpedance amplifiers. Equations (1) and (2) represent an ideal case for the determination of (x,y):

(1) $$x=\frac{Lx}{2}\frac{\left(V_{x2}+V_{y1}\right)-\left(V_{x1}+V_{y2}\right)}{V_{x1}+V_{x2}+V_{y1}+V_{y2}}$$

(2) $$y=\frac{Ly}{2}\frac{\left(V_{x2}+V_{y2}\right)-\left(V_{x1}+V_{y1}\right)}{V_{x1}+V_{x2}+V_{y1}+V_{y2}}$$

The function of the lens is to form an image of the beam of light emitted by the IR transmitters at points on the PSD sensor. The impact points, for image formation, and the SNR of the received signal will depend on the focal length and diameter of the lens, as does the field of view (FOV) and, therefore, the area covered by the PSD. We based our model of the system on the pin-hole model. Since we used an analog sensor, it was only possible to calculate the center of mass at the point of impact, as pixels were not available.

### 2.1. Linear Model

**Figure 3:**
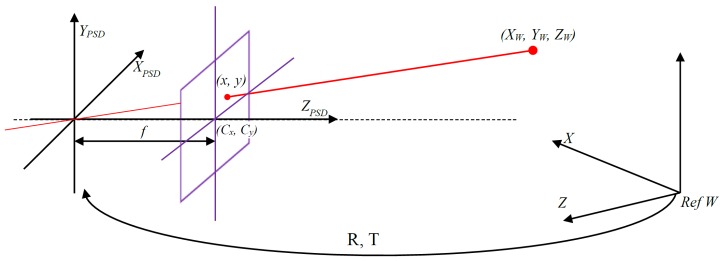
Pin-hole model.

The pin-hole model (Figure 3) represents an ideal optical system, which relates points in the environment to their corresponding points in the image plane, without taking distortions into account.

Expression (3) uses a rotation matrix (R) and a translation vector (T) to relate the world coordinate system with the optical coordinate system of the PSD sensor.

(3) $$\left(\begin{array}{c} X_{PSD}\\ Y_{PSD}\\ Z_{PSD} \end{array}\right)=R\left(\begin{array}{c} X_{W}\\ Y_{W}\\ Z_{W} \end{array}\right)+T$$

where $X_{W},Y_{W}$ and $Z_{W}$ are the coordinates of a point according to the world coordinate system, $X_{PSD},Y_{PSD}$ and $Z_{PSD}$ are the points according to the optical coordinate system and *R* and *T* are a 3 × 3 matrix and a 3 × 1 vector, respectively. The parameters in *R* and *T* are known as extrinsic parameters because they depend on the position and orientation between the world and the PSD sensor coordinate systems.

Lastly, the relationship between the optical reference system and the image is generated in the sensor image plane, according to Expression (4).

(4) $$\left(\begin{array}{c} s\cdot x\\ s\cdot y\\ s \end{array}\right)=\left(\begin{array}{ccc} f & 0 & C_{x}\\ 0 & f & C_{y}\\ 0 & 0 & 1 \end{array}\right)\left(\begin{array}{c} X_{PSD}\\ Y_{PSD}\\ Z_{PSD} \end{array}\right)$$

where $C_{x},C_{y}$ represents the optical center and *f* represents the focal length. These are the intrinsic parameters, which depend on the optical system. Besides, due to the perspective projection 3D to 2D, a scale factor *λ* is introduced into the equations. Thus, the entire geometrical model for the PSD sensor, based on the pin-hole model, is shown in Equation (5).

(5) $$\left(\begin{array}{c} s\cdot x\\ s\cdot y\\ s \end{array}\right)=\lambda \left(\begin{array}{ccc} f & 0 & C_{x}\\ 0 & f & C_{y}\\ 0 & 0 & 1 \end{array}\right)\left(\begin{array}{cccc} r_{11} & r_{12} & r_{13} & T_{x}\\ r_{21} & r_{22} & r_{23} & T_{y}\\ r_{31} & r_{32} & r_{33} & T_{z} \end{array}\right)\left(\begin{array}{c} X_{W}\\ Y_{W}\\ Z_{W} \end{array}\right)$$

where $r_{ij}$ are the parameters of the rotation matrix and $T_{x,y,z}$ are the parameters of the translation vector. A concise version of this expression is as follows.

(6) $$\left(\begin{array}{c} s\cdot x\\ s\cdot y\\ s \end{array}\right)=A\left(\begin{array}{cccc} r_{1} & r_{2} & r_{3} & t \end{array}\right)\left(\begin{array}{c} X_{W}\\ Y_{W}\\ Z_{W}\\ 1 \end{array}\right)=M\left(\begin{array}{c} X_{W}\\ Y_{W}\\ Z_{W}\\ 1 \end{array}\right)$$

where $r_{p=1,2,3}$ are the column vectors of *R*, matrix *A* is the intrinsic transformation and matrix *M* is the homography.

To the pin-hole model that represents the linear relationship between the environment and the image plane must now be added the effects produced by the lens.

### 2.2. Distortions

In our case, the most frequent and most important aberrations are radial and tangential distortions. We have the distortions of the lens and the distortion produced by the PSD sensor itself. The distortion of the PSD sensor depends on the type of PSD sensor, as explained in [17], which demonstrates that distortion with pin-cushion models may represent up to 1% of the size of the sensor. In our model, the center of distortion is located at the coordinate (0,0), whereas the center of lens distortion is located in the optical center $C_{x},C_{y}$, considering that these points probably do not coincide due to a bad coupling between the lens and the PSD sensor. The overlapping effects will present a complex behavior and interaction.

**Figure 4:**
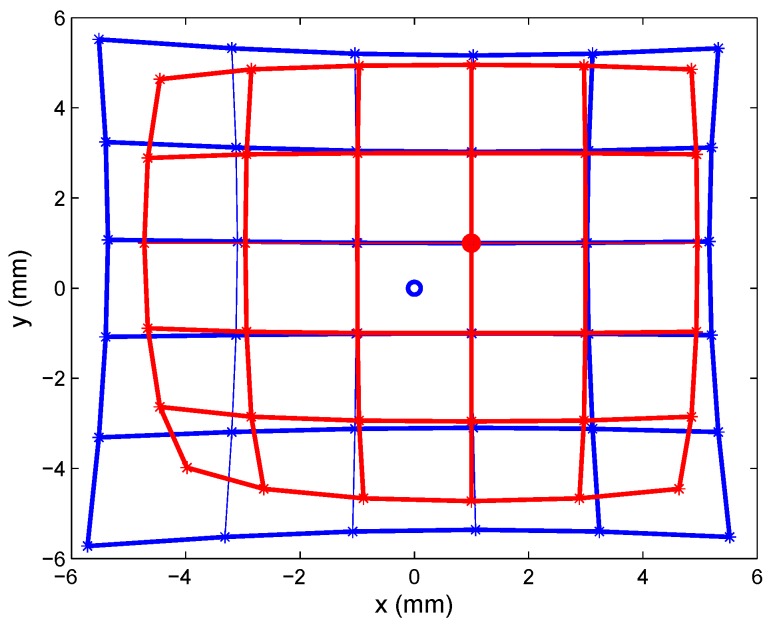
Blue: PSD sensor distortion; red: lens distortion; center in (1,1).

Figure 4a shows a possible simulation of both overlapped distortions, drawing in blue the PSD sensor distortion (centered at (0,0)) and in red the lens distortion with the distortion center (Cx=1,Cy=1).

The impact point $\left(x^{d},y^{d}\right)$, is the contribution of the distortion procedure by the lens and the PSD sensor; therefore, beginning with the PSD distortion, the expressions are:

(7) $$D_{x}^{Pr}=x^{d}\cdot \left(l_{1}h^{2}+l_{2}h^{4}+\cdots +l_{n}h_{n}^{n\cdot 2}\right)$$

(8) $$D_{y}^{Pr}=y^{d}\cdot \left(l_{1}h^{2}+l_{2}h^{4}+\cdots +l_{n}h_{n}^{n\cdot 2}\right)$$

where the parameter *h* is the Euclidean distance between the coordinates $\left(x^{d},y^{d}\right)$ and the coordinate origin, and the parameters that model the PSD sensor distortion are $l_{\left(i=1,2,\ldots ,m\right)}$. Therefore, the point that is distorted by the lens is:

(9) $$\left(x^{u}=x^{d}+D_{x}^{Pr}\right)$$

(10) $$\left(y^{u}=y^{d}+D_{y}^{Pr}\right)$$

Equations (11) and (12) represent the radial distortion by lens, which depends on the point $\left(x^{u},y^{u}\right)$:

(11) $$D_{x}^{lr}=\left(x^{u}-C_{x}\right)\cdot \left(k_{1}r^{2}+k_{2}r^{4}+\cdots +k_{n}r_{n}^{n\cdot 2}\right)$$

(12) $$D_{y}^{lr}=\left(y^{u}-C_{y}\right)\cdot \left(k_{1}r^{2}+k_{2}r^{4}+\cdots +k_{n}r_{n}^{n\cdot 2}\right)$$

where *r* is the Euclidean distance from the point $\left(x^{u},y^{u}\right)$ to the optical center $\left(C_{x},C_{y}\right)$, these considered as the center of the distortion, and $k_{\left(i,i=1,2,\ldots ,n\right)}$ are the parameters that model the radial lens distortion.

The expressions that model the tangential lens distortion are:

(13) $$D_{x}^{lt}=p_{1}\left(r^{2}+2{\left(x^{d}-C_{x}\right)}^{2}\right)+2p_{2}\left(x^{d}-C_{x}\right)\left(y^{d}-C_{y}\right)$$

(14) $$D_{y}^{lt}=p_{2}\left(r^{2}+2{\left(y^{d}-C_{y}\right)}^{2}\right)+2p_{1}\left(x^{d}-C_{x}\right)\left(y^{d}-C_{y}\right)$$

where $p_{1}$ and $p_{2}$ are the parameters that model tangential lens distortion.

### 2.3. Full Model

As was defined in the previous sections, the complete model, which relates the coordinate system of the environment with the coordinate system of the sensor, is the linear model obtained from model pinhole (Equation (5)), moreover the distortion corrections. Equations (15) and (16) represent the distortion corrections with two parameters for the lens distortion and one parameter for the PSD sensor.

(15) $$x=x^{d}+D_{x}^{Pr}+D_{x}^{lr}+D_{x}^{lt}$$

(16) $$y=y^{d}+D_{y}^{Pr}+D_{y}^{lr}+D_{y}^{lt}$$

where $D_{x}^{lr}$ and $D_{y}^{lr}$ are the radial distortion produced by the lens, $D_{x}^{lt}$ and $D_{y}^{lt}$ are the tangential distortion also produced by the lens and $D_{x}^{Pr}$ and $D_{y}^{Pr}$ are the radial distortion produced by the PSD sensor. As the radial distortions produced by lens and PSD sensor have the same expressions, in case the centers of the distortions are close to each other, the distortion corrections could be corrected together.

Having defined the mathematical model of our system, the next step was to develop a method by which we could calculate the parameters that would enable us to achieve our objective of accurately measuring the angle of arrival of a beam of light. Below, we describe the method developed for this purpose.

## 3. Calibration Process

There is no calibration method that is better than another, but depending on the type of camera, some methods give better results than others, so for example, in cases where the noise in the images is high, it is better to use methods based on 2D templates.

In our case, as mentioned in the Introduction, it is imperative that the calibration methods are based on 2D templates, because the LEDs should be directed to the PSD sensor so that the SNR of the signals in the PSD sensor is as high as possible, and therefore, the error in the calculation of the impact point is small. Therefore, of the methods based on 2D templates, Tsai, Batista and Zhang, the one that best adjusted to our needs is Zhang, since the calibration method of Tsai and Batista needs to know exactly certain parameters (optical center, focal length, etc.) to obtain the other parameters. This is a great handicap in our case, because we fix the optical system and the PSD sensor manually, and error in the measurement of these parameters would be high.

The calibration method selected for our system was an adapted version of Zhang’s method. This flexible method uses a 2D calibration pattern and permits the use of multiple images, being more robust to errors when capturing the template points. It has been shown to be a reliable method that yields very good results.

Zhang’s method requires the correspondences between points in space and their corresponding projections. The procedure consists of two stages. In the first step, a linear calculation is performed to obtain approximate values for linear system parameters, i.e., the mathematical model parameters excluding those that model distortions. In the second, nonlinear parameters are obtained by means of iterative methods, and the parameters calculated in the first stage are refined.

To improve the results obtained using the methods described and, therefore, the calibration models adapted to our needs, we incorporated proposals from other studies, such as [27,28], which suggest enhancing the results by performing a preliminary distortion correction and reducing image noise sensitivity, respectively.

As we will demonstrate later, preliminary distortion correction improves the results of the first stage of Zhang’s method, since this stage is very sensitive to image nonlinearities. However, when using lenses with low distortion, this correction process could be omitted.

These improvements will be analyzed in detail in Section 4, adapted and used in the model proposed in this paper.

Thus, the order of the calibration process proposed for our model was as follows: an initial rough distortion correction, followed by data normalization and then implementation of the proposed calibration method, which is an adapted version of Zhang’s method for PSD sensors. Each one of these processes is explained in the following subsections, in Section 3.4, to finally analyze error in the determination of the angle of arrival and mobile agent position.

### 3.1. System Calibration Method

The objective of this stage was to calculate the optical system parameter values, using nonlinear system algorithms (Gauss–Newton, Levenberg–Marquardt, gradient descent, etc.). One problem associated with these techniques is that they may converge on a solution that is mathematically correct, but that does not conform to reality. In order to avoid this situation, it is necessary to establish a good starting point, based on an approximate knowledge of the real parameter values or, in the case of Zhang’s method, by obtaining an approximation analytically.

To estimate the system’s linear parameter values for the starting point, Zhang performs two steps. In the first stage, the projection matrix (*M*) for each of the images is obtained using direct linear transformation (DLT), where Equation (17) must be solved by means of single value decomposition (SVD).

(17) $$\left(\begin{array}{c} s\cdot x\\ s\cdot y\\ s \end{array}\right)=M\left(\begin{array}{c} X_{t}\\ Y_{t}\\ Z_{t}\\ 1 \end{array}\right)$$

where x,y are the coordinates of the impact point in the sensor, *M* is a homography and $X_{t},Y_{t},Z_{t}$ the coordinates of corresponding point in the calibration template. As the calibration template is 2D the coordinate $Z_{t}$ is zero, Expression (18) relates the the calibration template points with the image plane points.

(18) $$\left(\begin{array}{c} s\cdot x\\ s\cdot y\\ s \end{array}\right)=A\left(\begin{array}{cccc} r_{1} & r_{2} & r_{3} & t \end{array}\right)\left(\begin{array}{c} X_{t}\\ Y_{t}\\ 0\\ 1 \end{array}\right)=A\left(\begin{array}{ccc} r_{1} & r_{2} & t \end{array}\right)\left(\begin{array}{c} X_{t}\\ Y_{t}\\ 1 \end{array}\right)$$

Knowing that $\begin{array}{ccc} {}[m_{1} & m_{2} & m_{3}] \end{array}=A\begin{array}{ccc} {}[r_{1} & r_{2} & t] \end{array}$, where $m_{i}$ are the columns of matrix M,A is the matrix containing the intrinsic parameters, $r_{i}$ are the columns of the rotation matrix and *t* is the translation vector, we know that since the rotation matrix is orthonormal, the following relationships can be applied: $r_{1}^{T}r_{1}=r_{2}^{T}r_{2}$ and $r_{1}^{T}r_{2}=0$. Consequently, from these constraints, we obtain Equations (19) and (20).

(19) $$m_{1}^{T}A^{-T}A^{-1}m_{2}=0$$

(20) $$m_{1}^{T}A^{-T}A^{-1}m_{1}=m_{2}^{T}A^{-T}A^{-1}m_{2}$$

where $A^{-T}A^{-1}$ results in the matrix *B* given in Equation (21), which is the proposal for our model, where the focal length parameter is considered of equal value for the two coordinate axes.

(21) $$B=A^{-T}A^{-1}=\left(\begin{array}{ccc} 1/f^{2} & 0 & -C_{x}/f^{2}\\ 0 & 1/f^{2} & -C_{y}/f^{2}\\ -C_{x}/f^{2} & -C_{y}/f^{2} & C_{x}^{2}/f^{2}+C_{y}^{2}/f^{2}+1 \end{array}\right)=\left(\begin{array}{ccc} b_{11} & b_{12} & b_{13}\\ b_{21} & b_{22} & b_{23}\\ b_{31} & b_{32} & b_{33} \end{array}\right)$$

As the matrix *B* is symmetrical, it is only necessary to obtain six elements, so the vector is $b=[\begin{array}{cccccc} b_{11} & b_{12} & b_{22} & b_{13} & b_{23} & b_{33}{]}^{T} \end{array}$. The elements of matrix B are traditionally obtained using Equation (22), where, $V_{ij}=\left(\begin{array}{cccccc} m_{i1}m_{j1} & m_{i1}m_{j2}+m_{i2}m_{j1} & m_{i2}m_{j2} & m_{i3}m_{j1}+m_{i1}m_{j3} & m_{i3}m_{j2}+m_{i2}m_{j3} & m_{i3}m_{j3} \end{array}\right)$ and $m_{ij}$ are the elements of matrix *M* obtained by DLT.

(22) $$\left[\begin{array}{c} V_{12}^{T}\\ {\left(V_{11}-V_{22}\right)}^{T} \end{array}\right]b=0$$

By retrieving the vector *b*, the intrinsic parameters are then obtained with the following expressions:

(23) $$C_{x}=-b_{11}/b_{13}$$

(24) $$C_{y}=-b_{22}/b_{23}$$

(25) $$f=\sqrt{1/b_{11}}$$

Once the intrinsic parameters have been calculated, the projection matrices for each image are used to calculate the extrinsic parameters as follows:

(26) $$r_{1}=\lambda A^{-1}m_{1}$$

(27) $$r_{2}=\lambda A^{-1}m_{2}$$

(28) $$t=\lambda A^{-1}m_{3}$$

where $\lambda =∥\frac{1}{A^{-1}m_{1}}∥=∥\frac{1}{A^{-1}m_{2}}∥$. Due to image noise and nonlinearity, these parameters contain error because we are using linear techniques to solve a nonlinear problem. Hence, in Section 3.2, we present a prior image distortion correction, which improves the results.

Having obtained approximate linear parameter values, the extrinsic and intrinsic parameters that model the system are obtained/optimized, in our case using the Levenberg–Marquardt algorithm.

The cost function used in [25] minimizes error in the projection of the points on the image plane. The proposed cost function for our model, and the one with which we obtained the best results, minimizes the errors of the points on the calibration template (distance between points on the template and projection of the image points onto the template) and is expressed as follows:

(29) $$\sum_{i=1}^{n}\sum_{j=1}^{m}{∥P_{j}-\hat{P_{j}}\left(A,D,R_{i},T_{i},Q\right)∥}^{2}$$

where *n* is the number of images, *m* is the number of points per image, *Q* are points in the image plane, *A* is the intrinsic matrix containing the parameters, such as the focal length and optical center, vector *D* contains the parameters that model distortion, $R_{i}$ and $T_{i}$ are the rotation matrix and the translation vector, respectively, $\hat{P_{j}}$ are the calibration template points and *P* are the re-projected image plane points (points obtained from the intrinsic and extrinsic parameters and the image plane points).

### 3.2. Preliminary Distortion Correction

As mentioned earlier, this step is essential in the case of high distortion lenses. Of the several methods described and proposed in [27] to reduce image distortion, we selected the “line straightness method”. With regard to PSD sensor distortion, we assumed that it would exert little effect on image distortion since it would be considerably less than the distortion generated by the lens. Nevertheless, it will also be analyzed in the following sections.

In this section, we describe how to estimate the coefficients of the distortion produced by the lenses in order to correct the distortion. In our case, we did this to improve the results obtained in the first stage of Zhang’s method, which is very sensitive to nonlinearity. Estimation of those coefficients is based on analyzing curves, which are actually straight lines that have been distorted by the lens, using iterative methods.

An example is given in [27], in which one radial distortion coefficient and one tangential distortion coefficient are estimated; however, we estimated two radial distortion coefficients since in the majority of cases, tangential distortion exerted less influence.

The method is based on detecting the lines found in the image, which in our case were easy to identify since we used a calibration template, and then obtaining the slope at several points along the lines. If they were straight, the points along a line would have the same slope. However, due to lens distortion, this is not the case, and therefore, the cost function to minimize is the slope of the tangents at each of the points on the line.

Hence, the cost function to minimize is as follows:

(30) $$\sum_{j=1}^{L}\sum_{i=2}^{N}{∥s_{j}\left(x_{i}^{d},y_{i}^{d}\right)-s_{j}\left(x_{i-1}^{d},y_{i-1}^{d}\right)∥}^{2}$$

where *L* is the number of lines, *N* is the number of points on each line, $\left(x^{d},y^{d}\right)$ are the coordinates of each point and *s* is the slope at that point.

(31) $$s\left(x^{d},y^{d}\right)=\frac{\frac{\partial x^{u}}{\partial x^{d}}+\frac{\partial x^{u}}{\partial y^{d}}\frac{\delta y^{u}}{\delta x^{d}}}{\frac{\partial y^{u}}{\partial x^{d}}+\frac{\partial y^{u}}{\partial y^{d}}\frac{\delta y^{u}}{\delta x^{d}}}$$

where $\frac{\delta y^{d}}{\delta x^{d}}$ is the slope of the tangent at the point $\left(x^{d},y^{d}\right)$ and the partial derivatives for estimating the radial distortion coefficients are as follows:

(32) $$\frac{\partial x^{u}}{\partial x^{d}}=k_{1}\left(r^{2}+2\left(C_{x}-x^{d}\right)\left(C_{x}-x^{d}\right)\right)+4k_{2}\left(r^{2}{\left(C_{x}-x^{d}\right)}^{2}+r^{4}\right)+1$$

(33) $$\frac{\partial y^{u}}{\partial y^{d}}=k_{1}\left(r^{2}+2\left(C_{y}-y^{d}\right)\left(C_{y}-y^{d}\right)\right)+4k_{2}\left(r^{2}{\left(C_{y}-y^{d}\right)}^{2}+r^{4}\right)+1$$

(34) $$\frac{\partial x^{u}}{\partial x^{d}}=2k_{1}\left(C_{y}-y^{d}\right)+4k_{2}r^{2}\left(C_{y}-y^{d}\right)\left(C_{x}-x^{d}\right)$$

(35) $$\frac{\partial x^{u}}{\partial x^{d}}=2k_{1}\left(C_{x}-x^{d}\right)+4k_{2}r^{2}\left(C_{y}-y^{d}\right)\left(C_{x}-x^{d}\right)$$

where *r* is the Euclidean distance between point $\left(x^{d},y^{d}\right)$ and the optical center $\left(C_{x},C_{y}\right)$. In the simulations section, we will analyze the influence of this preliminary distortion correction when obtaining the linear values in the first stage of Zhang’s method, which will serve as the starting point.

### 3.3. Data Normalization

The matrix *M* that relates scene points to their projection on the image plane is sensitive to image plane position and image noise, as given in [28]. To resolve this problem and obtain more stable results, we proposed to obtain a better conditioned projection matrix than the one obtained by DLT. Thus, starting from Equation (36):

(36) Q=MP

where $Q_{3xn}$ is the matrix with the impact points for each image, $M_{3x3}$ is the homography and $P_{3xn}$ are the template points. Our proposal is to normalize matrix *Q* by a matrix $T_{Q3x3}$ and the template points by a matrix $T_{P3x3}$, obtaining Equation (37).

Being $T_{Q}$ and $T_{p}$ created under the following conditions, the calibration template and its projection on the image plane must be centered on the coordinate origin, and the points must be isotropically scaled, so that the mean distance to the origin is equal to $\sqrt{2}$; obviously, $T_{p}$ always is the same matrix because the coordinates of the template do not change. Thus, we have:

(37) $$T_{Q}Q=M^{o}T_{P}P$$

where $M^{o}$, which relates $T_{Q}Q$ to $T_{P}P$, is obtained by DLT.

Having obtained $M^{o}$, all that remains is to solve Equation (38) in order to obtain the real homography.

(38) $$M=T_{Q}^{-1}M^{o}T_{p}$$

Next, Section 3.4 describes an analysis of the sensibility of AoA, due to the impact point and focal length.

### 3.4. Analysis of AoA Uncertainty Regarding Optical System Parameters

**Figure 5:**
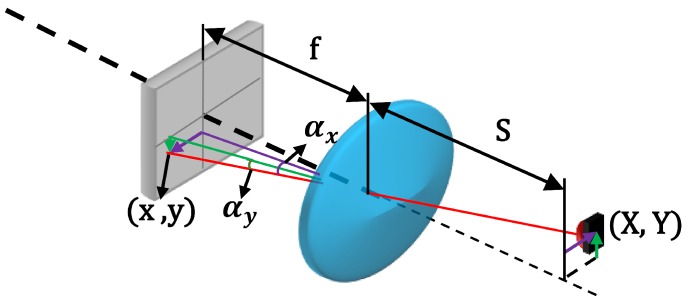
PSD attached to an optical lens.

In this section, we describe the sensitivity of the measurement system to errors in the optical system parameters, to demonstrate the robustness or weakness. Based on Figure 5, the equation to obtain the angle of arrival on the *x* axis is given by Expression (39); a similar expression and conclusions may be extracted for the *y* axis.

(39) $$\alpha_{x}=arctan\frac{x}{f}$$

where $\alpha_{x}$, $\alpha_{y}$ represent the angles of arrival, (x,y) is the impact point, (X,Y) is the position of the IR transmitter, *S* is the distance between the optical system and the IR transmitter and *f* is the focal length.

The partial derivatives to determine the error in the angle of arrival due to each of the intrinsic parameters are as follows:

(40) $$\varepsilon_{\alpha x}=\left|\frac{\partial \alpha_{x}}{\partial x}\right|\Delta_{x}=\left|\frac{f}{f^{2}+x^{2}}\right|\Delta_{x}$$

(41) $$\varepsilon_{\alpha f}=\left|\frac{\partial \alpha_{x}}{\partial f}\right|\Delta_{f}=\left|\frac{x}{f^{2}+x^{2}}\right|\Delta_{f}$$

The final expression of the error in the angle of arrival is as follows:

(42) $$\varepsilon_{\alpha }=\left|\frac{\partial \alpha_{x}}{\partial x}\right|\Delta_{x}+\left|\frac{\partial \alpha_{x}}{\partial f}\right|\Delta_{f}$$

**Figure 6:**
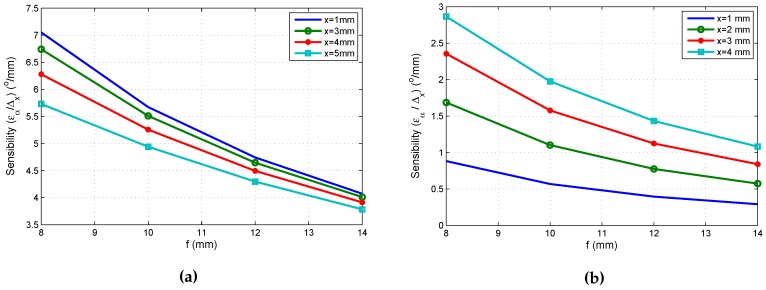
(**a**) Sensibility of the angle due to error in the impact point; (**b**) sensibility of the angle due to error in the focal length.

Figure 6a,b show the sensitivity with respect to the impact point and focal length.

Comparing Figure 6a,b, one can show that the sensibility due to error in the impact point is higher than the error coming from the focal length.

## 4. Evaluation of the Proposed Model and Calibration Method

In what follows, we evaluate the accuracy and sensitivity to different effects of the proposed calibration method. To this end, we describe four tests. The first of these assesses the improvements yielded by the proposed preliminary distortion correction and data normalization. The second assesses the effect of PSD sensor signal noise, using different levels of SNR, which is added to the output signal of the amplifiers. The third test evaluates the effect of imbalances in amplifier gain factors alone (without noise) and in combination with the effect of noise. The fourth and final test assesses PSD sensor distortion, which is also analyzed alone and in combination with noise and imbalances. To conclude, the results are compared with different sources of error.

### 4.1. Tests Setup

The different tests have considered two types of lens to verify that the proposed calibration method is robust in either case. Since distortion is an important problem, we evaluated the calibration using one lens with low distortion (long focal length) and another with higher distortion (short focal length):

- Lens 1: focal length of 25 mm and one radial distortion parameter ($k_{1}=2\;\times \;10^{-3}$ mm).
- Lens 2: focal length of 8 mm and two radial distortion parameters ($k_{1}=1\;\times \;10^{-2}$, $k_{2}=3\;\times \;10^{-5}$ mm).

**Figure 7:**
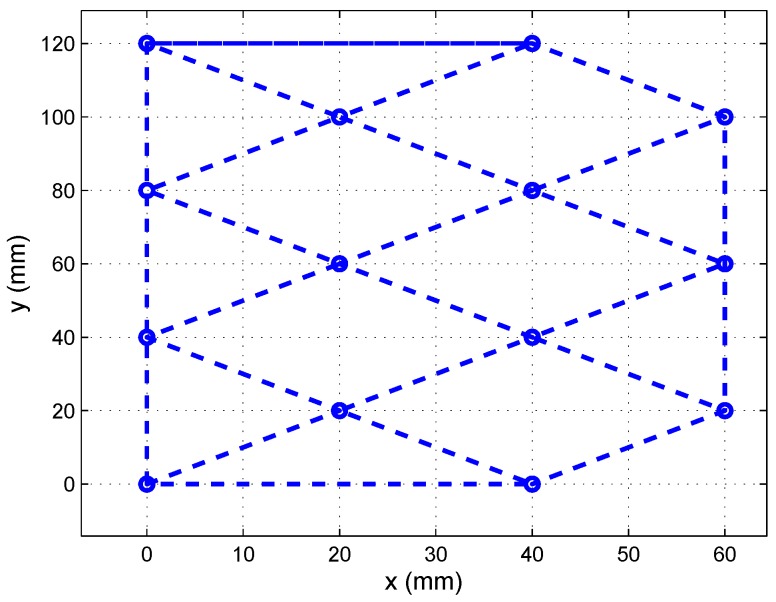
Calibration template.

The tests were performed using synthetic images created from the calibration template shown in Figure 7. The points in the calibration template were unaligned to enhance the calculation of the projection matrix.

**Figure 8:**
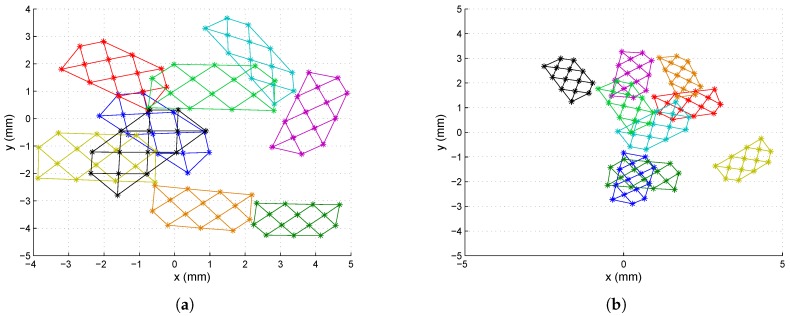
Calibration set of images: (**a**) set images of Lens 1; (**b**) set of images of Lens 2.

Figure 8a shows Set 1 of the synthetic images (nine images for each lens), which were captured using a 25-mm focal length lens, placing the calibration template in different positions. Figure 8b shows a set of images captured with an 8-mm focal length lens (note that the template was not placed in the same positions in each case). In both cases, the images were generated with a misalignment from the optical center with a displacement of (0.5, −0.7) mm with respect to the electrical center. The extrinsic parameter values used to perform the test were randomly selected.

As shown in Figure 8, the images captured should cover the whole PSD surface sensor to produce a good distortion correction.

### 4.2. Evaluation of the Preliminary Corrections

First, we will evaluate the preliminary distortion correction and data normalization by comparing the intrinsic parameter values obtained in the first calibration stage, which yielded the initial values for the iterative method. Table 1 and Table 2 show the results for Lenses 1 and 2, respectively. We used Set 1 and Set 2, each one with nine ideal images, i.e., without noise or imbalances in the signal gain factors and without taking PSD sensor distortion into account (these sources of error will be evaluated in the following sections).

The best results were obtained when using preliminary distortion correction, whereas any improvement due to data normalization was imperceptible, perhaps because of the lack of noise in the images. In the following subsection, we discuss a simulation of the calibration method using the same images, but with noise.

### 4.3. Evaluation of the Effect of PSD Sensor Signal Noise on the Calibration Process

To evaluate the effect of noise in the PSD sensor output signals on geometric calibration, we conducted tests with SNR values of between 30 and 50 dB, adding white Gaussian noise with a distribution of $\left(N\left(\mu ,\sigma^{2}\right)\right)$. Having introduced noise into the signals, these were filtered before calculating the point of impact on the PSD sensor surface. We used a Butterworth bandpass filter with a 600-Hz bandwidth centered at a frequency of 1 kHz.

The calibration results also depend on other variables, such as the number of images, lens parameters and the images captured with each lens (position of the images in the image plane).

We used nine images for the two lenses in the calibration simulations and also included or omitted the preliminary distortion correction and improved projection matrix calculation (indicated in the following tables as “with improvements”), to compare the effect of these improvements. We performed 200 different test instances and extract the mean and std values.

Table 3 shows the results of calibration for Lens 1, giving the ideal and the measured intrinsic parameter values, as well as the mean and standard deviation in parenthesis.

The results obtained indicate that there are few differences in the results for the intrinsic parameters; however, when the improvements were incorporated, the iterative method converged on fewer iterations, and the starting point obtained in these cases was closer to the final solution. One indication that the calibration results were good are the residuals. These indicate the error between the template points and the image points re-projected into the environment, i.e., the points in the image plane translated to the environment using the parameter values obtained in the calibration, where the residuals are the sum of the error in each of the points in each image.

Table 4 shows the results for the simulation with Lens 2, which had a shorter focal length than the previous lens and higher distortion. As in the previous case, 200 iterations were performed for each SNR level, and the simulation was also carried out with and without preliminary distortion correction and data normalization.

The results were similar to those for the previous simulation. The number of iterations was significantly lower when the preliminary corrections were incorporated because the distortion was higher, and therefore, the images were less linear and the projection matrix calculation worse.

These simulations indicated that preliminary distortion correction and data normalization improve the calibration process, and they were therefore incorporated in the following tests.

### 4.4. Evaluation of the Effect of Imbalance in Amplifier Gain Factors on the Geometric Calibration

Another source of error is imbalance in gain factors, i.e., differences between the electronic components involved in gain factor values. To perform the simulation, we selected tolerances of 1% and 15% for the resistor and capacitor, respectively, with nominal values of 100 kΩ and 100 pF, where the equation for the transimpedance amplifier gain factor is as follows:

(43) $$K=\frac{R}{RC2\pi f+1}$$

This factor also depends on the *R* and *C* values of the working frequency, where the greatest error is produced at 13.793 kHz, as described in [15]; the nominal gain factor at this frequency is 53,572, and variation in this factor with the above-mentioned tolerances is 4017.9.

Variation in the gain factor for each signal was simulated assuming that distribution was uniform U[a,b]. As before, we also performed tests with white Gaussian noise $\left(N\left(\mu ,\sigma^{2}\right)\right)$.

For each case, 200 iterations were performed, obtaining the results shown in Table 5 and Table 6 for Lenses 1 and 2, respectively, giving the mean value and standard deviation in brackets for the intrinsic parameters.

The results obtained indicate that when the electrical components involved in signal gain were not precision components, the intrinsic parameters obtained for the optical system were incorrect, with large errors in the optical center $\left(C_{x},C_{y}\right)$ and somewhat smaller errors in the other parameters (focal length and distortions). The results obtained with and without adding noise (SNR of 40–50 dB) to the measurements were almost identical.

The results for Lens 2 were similar to those for Lens 1 in that the images with and without noise were approximately the same, and thus, the predominant error was that due to imbalances. Consequently, as discussed in [15], it is necessary to calibrate the amplification step, since this could give rise to unacceptable errors in geometric calibration.

### 4.5. Analysis of Pin-Cushion PSD Sensor Distortion

Here, we will analyze the effect of PSD sensor distortion, an error that is characteristic of the type of PSD sensor employed. For our tests, we used the Hamamatsu PSD S5991-01 sensor, a pin-cushion type sensor in which, as explained in [17], distortion is approximately 1% of the size of the PSD sensor and where the center of distortion is the center of the PSD sensor (0,0). This pin-cushion type distortion is the opposite of the distortion produced by the lenses that we used in our system, which are biconvex and produce barrel-type distortion, suggesting that if the optical center coincides with or is very close to the electrical center of the PSD sensor, the contribution of this distortion to error should be small. However, if these two centers do not coincide, it is possible that the intrinsic system parameters will be affected. The PSD sensor distortion was modeled as a radial distortion with a single parameter value of $-1.3058\;\times \;10^{-3}$.

As in the previous cases, the simulations were performed with two lenses under three conditions: with the effect of PSD sensor distortion alone, with PSD sensor distortion plus imbalances and in combination with white Gaussian noise $\left(N\left(\mu ,\sigma^{2}\right)\right)$ at an SNR value of 40 dB.

Table 7 shows the intrinsic parameter results for Lens 1 under all the conditions described above, giving the mean value and standard deviation in brackets.

Note that in this test, the parameter $k_{1}$ is the contribution of lens distortion and PSD sensor distortion; therefore, the ideal value is 6.9416$\;\times \;10^{-4}$.

The results obtained indicate that when distortion is 1% of the size of the PSD sensor and images are contaminated with noise, the errors are acceptable. In the case of higher distortion, this could be corrected by disconnecting the lens and scanning the entire PSD sensor surface, obtaining the distortion of the PSD sensor itself and correcting the images prior to the calibration process.

Table 8 shows the results for Lens 2 under the same conditions as those used for Lens 1.

As in the previous case, $k_{1}$ is the contribution from the lens distortion and PSD sensor distortion, the ideal value being 8.69415$\;\times \;10^{-3}$.

### 4.6. Conclusions Drawn from the Simulations

Here, we will compare the results obtained for the different simulations. The results obtained for Lens 1 are compared in Table 9 and those for Lens 2 in Table 10.

As expected, the results confirm that the worst case was when all of the effects were added. However, it can be seen that gain imbalances constituted the greatest source of error, and as has been mentioned, these can be corrected [15]. Therefore, the most realistic case would be that of noise and PSD sensor distortion.

PSD distortion is constant, and thus, if the distribution of captured images is judiciously selected, its effect can to a large extent be corrected implicitly when lens distortion is corrected.

In terms of noise, signal SNR may be greater than 50 dB for the calibration process; however, the results obtained with an SNR of 50 dB and PSD sensor distortion were very close to ideal. In addition, the more images used, the better the results.

**Figure 9:**
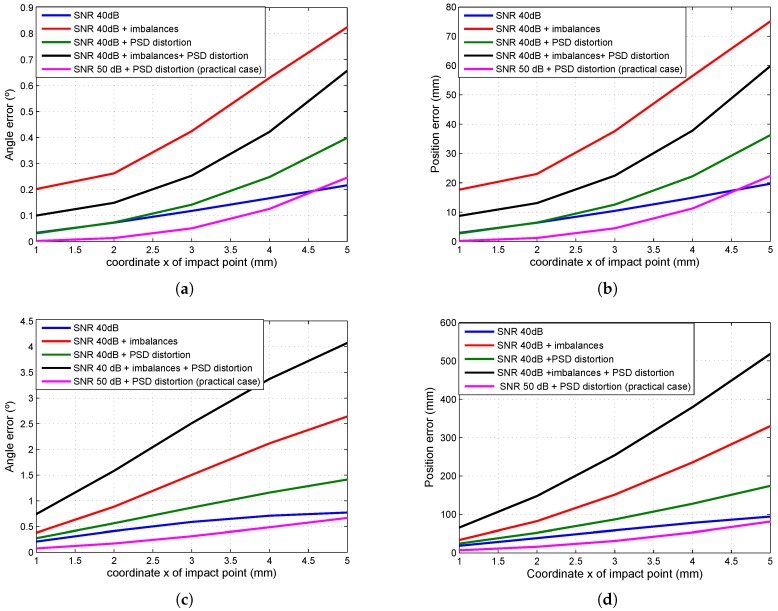
(**a**) Error in the AoA for Lens 1; (**b**) error in mobile agent position determination for Lens 1; (**c**) error in the AoA for Lens 2; (**d**) error in mobile agent position determination for Lens 2.

Figure 9 shows the errors in the angles of arrival and in position determination depending on the point of impact of the beam, where the distance between the plane in which the mobile agent is moving and the detector is 5 m. The graphs were generated using the results shown in Table 9 and Table 10, while the parameter values employed were the mean values plus twice the value of the standard deviation.

The best result was obtained with an SNR of 50 dB and PSD distortion and was very similar to that obtained when solely taking noise with an SNR value of 40 dB into account. In both cases for Lens 1, the error in mobile agent position determination was around 2 cm, while in the case of Lens 2, it was less than 10 cm (when furthest from the PSD).

As the graphs show, the error in the tests that included the gain imbalances’ factor is approximately four-times higher.

In conclusion, the proposed calibration method (preliminary distortion correction, data normalization and Zhang’s method adapted to small, continuous sensors) yielded good results in cases without gain factor imbalances and when the SNR was >40 dB, regardless of lens and/or PSD sensor distortion. In the worst case for Lens 1, the error in the angle of arrival and mobile agent position determination was around 0.25°, and for Lens 2, it was 0.7°.

These simulations were performed to analyze the most problematic sources of error, but there are other sources of error that were not modeled and which arose in the experimental tests, such as a poor connection between the lens and the PSD sensor, a faulty and/or asymmetrical lens or manufacturing problems with the PSD sensor.

## 5. Experimental Tests

**Figure 10:**
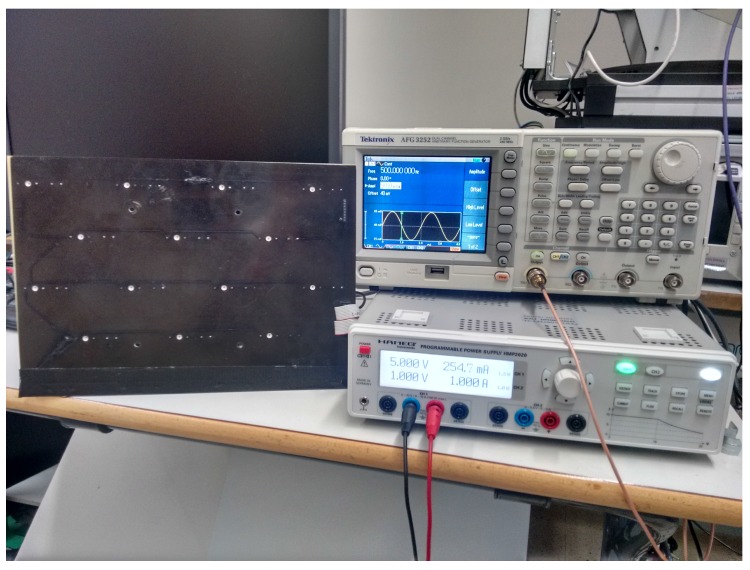
Calibration template and signal generation instruments.

For these tests, we used a 2D calibration template consisting of 14 OSRAM SFH-4233 LEDs, as shown in Figure 10. A National Instruments PCI-6289 data acquisition card was used to control data acquisition and to switch the LEDs on and off. The outgoing signal was a 1000-Hz sinusoidal signal generated with an AFG 3022B-Tektronix. The frequency was selected bearing in mind the harmonics of other sources of noise (mains electricity, fluorescent lighting).

**Figure 11:**
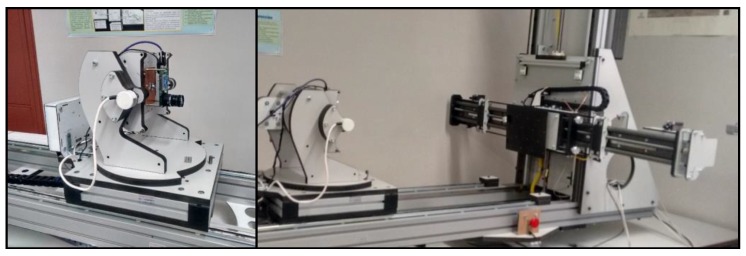
Automated mounting.

To obtain the different images (Figure 11), an automated system was used with five degrees of freedom, three of translation and two of rotation.

The first calibration was performed with a 40-mm focal length lens measuring 1 in in diameter; given the large focal length, radial distortion and the field of view angle were low. The second calibration was performed with a spacecom JF7.5M-2 lens with a focal length of 7.5 mm and a field of view of 50°, yielding appreciable radial distortion.

**Figure 12:**
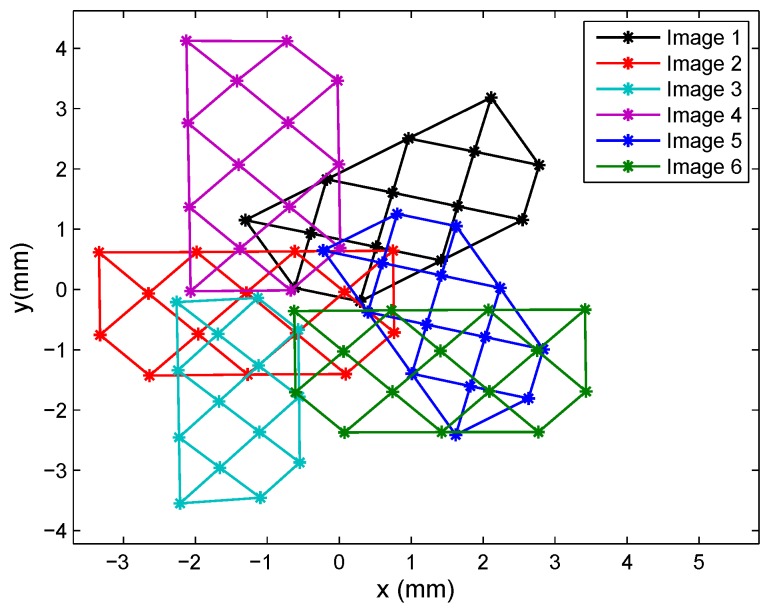
Images for Lens 1.

**Figure 13:**
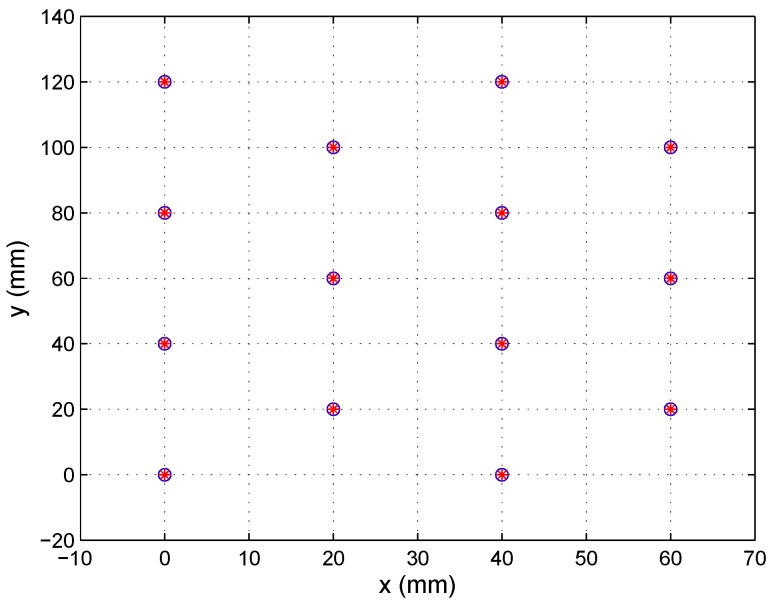
Residuals.

In the first test, we used 12 images covering the entire PSD sensor surface, in different positions and angles, six of which are depicted in Figure 12 (not all of the images have been included, as they overlap and would be difficult to distinguish if the entire set were shown in the figure). Table 11 shows the results obtained for the intrinsic parameters using a different number of images, in this case, the results of 8–12 images. The first row with the legend “residuals” refers to the sum of errors in all images between points in the calibration template and re-projected points (from the images in the PSD onto the template), using the parameter values obtained during calibration. In Figure 13, the error using 12 images is depicted.

As can be seen, the results remained stable with small changes from 10 images onward, and therefore, the result will remain unchanged even if more images are used. Note that for each image, the residuals are the sum of errors of: 14 points · number of images.

**Figure 14:**
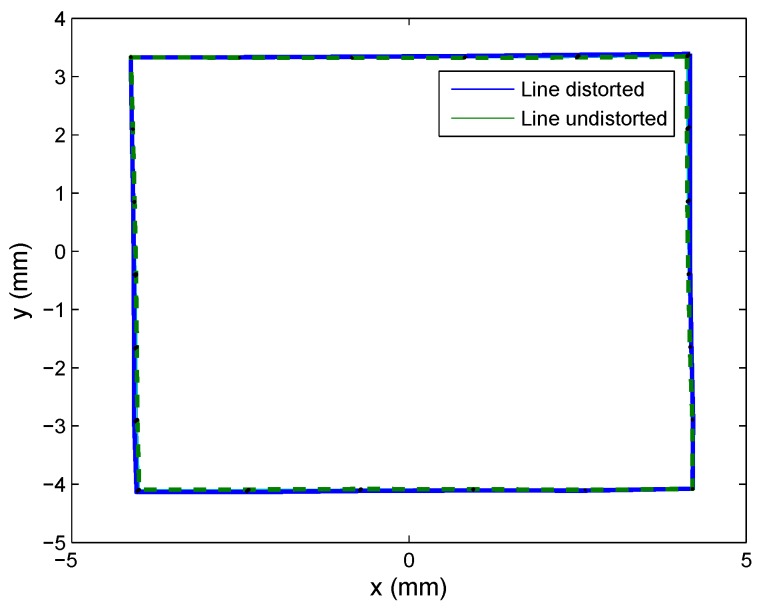
Distorted points vs. corrected points.

To determine whether optimization of the calibration process generated intrinsic parameters of similar dimensions to those of the real parameters (abstraction of the method could yield mathematically-correct solutions that would solve the calibration, but with values that did not conform to the real parameters), we examined, by way of example, the focal length value. For this, we took four points from those shown in Figure 14 on the PSD sensor (the blue line represents measured points and the green line corrected points; note that this is a lens without distortion).

Given the coordinates for the points (x,y) and the distance from the PSD sensor to the transmitter, we calculated the focal length using Equation (44).

(44) $$f=\frac{H}{h}S$$

where *f* is the focal length, *H* is the distance between two points in the environment, *h* is the distance between the same points, but in the image plane, and *S* is the distance between the optical system and the transmitter.

Using the corners of the square in Figure 14, where *S* is equal to 950 mm ± 1 mm (error caused by the automated mounting encoder), we found that:

- From the lower right corner to the lower left corner, His equal to 200 mm and h is equal to 8.2058 mm; therefore, the focal length is 38.9776 mm.
- From the lower left corner to the upper left corner, H is 180 mm and h is 7.4285; therefore, the focal length is 39.2060 mm.
- From the upper left corner to the upper right corner, H is 200 mm and h is 8.2456; therefore, the focal length is 39.1666 mm.
- From the upper right corner to the lower right corner, H is 180 mm and h is 7.4297 mm; therefore, the focal length is 39.2123 mm.

As can be seen, these are within range of the solution given by calibration.

**Figure 15:**
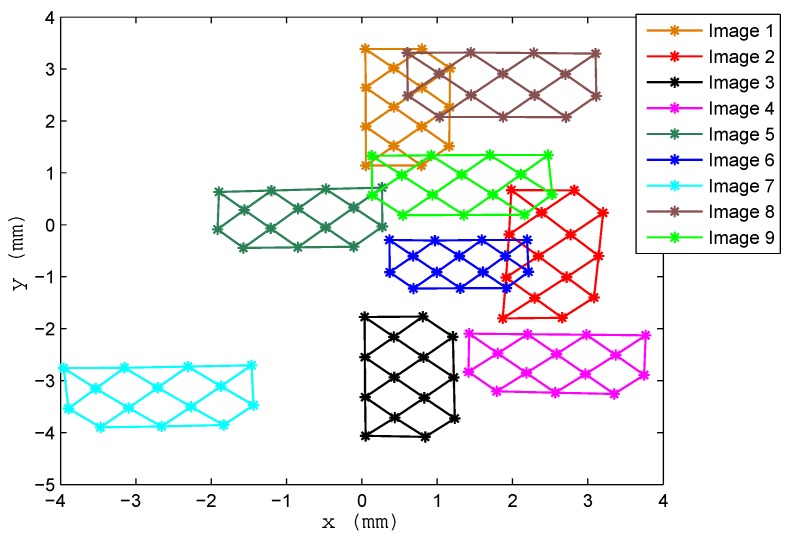
Images for Lens 2.

In the case of calibration using the JF7.5M-2 lens, we also used 12 images, six of which are shown in Figure 15.

**Figure 16:**
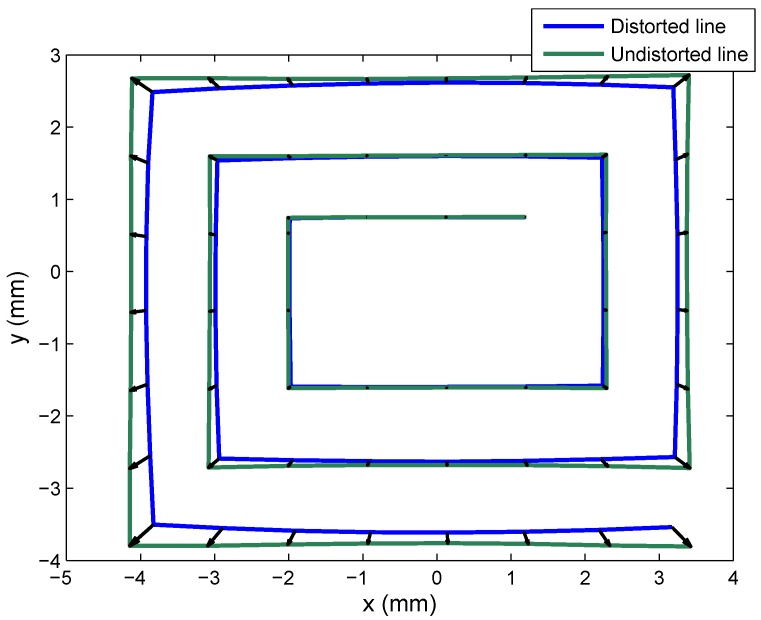
Correction of Lens 2 distortion.

To provide a visual depiction of how distortion was corrected, we took points from the entire surface of the PSD sensor equipped with the JF7.5M-2 lens. The test mounting described earlier was used to perform linear movements forming a “spiral”. These data are shown in Figure 16, where the blue line shows the points obtained with clearly evident radial distortion, and the green line shows the points corrected with the intrinsic parameter values (distortion and optical center) obtained from calibration.

We also conducted the same test with Lens 2, to calculate the focal length according to Equation (44). Taking the points at the very outer corners of the “spiral” and given that the distance between the PSD sensor and the IR transmitter was 350 mm, we found that:

- From the lower right corner to the lower left corner, H is equal to 350 mm and h is equal to 7.5714 mm; therefore, the focal length is 7.5714 mm.
- From the lower left corner to the upper left corner, H is 300 mm and h is 6.4663; therefore, the focal length is 7.5441 mm.
- From the upper left corner to the upper right corner, H is 350 mm and h is 7.5130; therefore, the focal length is 7.5130 mm.
- From the upper right corner to the lower right corner, H is 300 mm and h is 6.4797 mm; therefore, the focal length is 7.5597 mm.

In Table 12, which shows the parameter values obtained from calibration, the focal length was 7.4933 mm, while in the results obtained in this test, the greatest difference from this figure was 0.0781 mm, indicating that the calibration results were close to real values.

## 6. Conclusions

The simulations and empirical tests demonstrated that the best system calibration results were obtained when using the proposed model and calibration procedure, which commences with a preliminary distortion correction, since the calibration method is very sensitive to lens distortion. However, we found that in contrast to reports in other studies, data normalization results were no different to those obtained using DLT. We also found that in order to calibrate the system, signal noise must be low (SNR > 40 dB), although this does not present a problem, since calibration can be performed over short distances and, therefore, with high signal levels.

Another source of error that can be disregarded is PSD sensor distortion in the event that it is a pin-cushion sensor, since this type of PSD presents low distortion that is canceled out by calibrating lens distortion.

The larger error is produced by errors in the gain factors. Our results indicate that these could produce the largest errors since they depend on the quality of the components; thus, it would be necessary to perform an electrical calibration as explained in [15], to eliminate this source of error.

As regards the experimental tests, the results obtained were close to reality, as shown in the focal length calculation using Equation (44), which coincides with the focal length obtained in the geometric calibration, for two lenses. This also indicates that distortion was corrected in the case of the second lens, since if it were not duly corrected, the error in parameter (*h*) would generate a very different focal length value from that obtained from calibration.

Correction of the distortion produced by the second lens is depicted in Figure 16.

## Figures and Tables

**Table 1:** Comparison of the intrinsic parameter values obtained in the first calibration stage.

Lens 1	Ideal	Without Preliminary Distortion Correction	Without Preliminary Preliminary Distortion and Data Normalization	With Distortion Correction Correction	With Preliminary Distortion Correction and Data Normalization
$C_{x}$ (mm)	0.5	0.33172	0.31600	0.3589	0.2871
$C_{y}$ (mm)	−0.7	−1.5623	−1.5030	−1.3373	−1.3077
*f* (mm)	25	26.2146	25.7406	25.1194	25.1559

**Table 2:** Comparison of the intrinsic parameter values obtained in the first calibration stage.

Lens 2	Ideal	Without Preliminary Distortion Correction and DLT	Without Preliminary Distortion Correction and Data Normalization	With Preliminary Distortion Correction and DLT	With Preliminary Distortion Correction and Data Normalization
$C_{x}$ (mm)	0.5	0.5017	0.6585	0.5103	0.5113
$C_{y}$ (mm)	−0.7	−0.7370	−0.6160	−0.7520	−0.7095
*f* (mm)	8	10.4352	11.0650	9.2115	9.1549

**Table 3:** Evaluation of the calibration method with electrical signal noise.

Lens 1		With Corrections			Without Corrections		
SNR (dB)	Ideal	30	40	50	30	40	50
$C_{x}$ (mm)	0.5	0.6581	0.5062	0.5060	0.7700	0.5097	0.4981
		(0.7162)	(0.1551)	$\left(4.3929\;\times \;10^{-2}\right)$	(0.6315)	(0.1665)	$\left(4.1426\;\times \;10^{-2}\right)$
$C_{y}$ (mm)	−0.7	−0.7749	−0.6806	−0.6920	−0.2968	−0.6844	−0.7002
		(0.5186)	(0.2219)	$\left(7.2875\;\times \;10^{-2}\right)$	(0.6589)	(0.2574)	$\left(7.8348\;\times \;10^{-2}\right)$
*f* (mm)	25	24.9084	24.9868	24.9954	24.8869	24.9951	25.0009
		(0.4832)	(0.1724)	$\left(5.4405\;\times \;10^{-3}\right)$	(0.5935)	(0.1683)	$\left(4.7079\;\times \;10^{-2}\right)$
$k_{1}$ (mm)	2$\;\times \;10^{-3}$	2.0140$\;\times \;10^{-3}$	1.9962$\;\times \;10^{-3}$	2.0014$\;\times \;10^{-3}$	1.9207$\;\times \;10^{-3}$	1.9957$\;\times \;10^{-3}$	1.9961$\;\times \;10^{-3}$
		$\left(2.0461\;\times \;10^{-4}\right)$	$\left(4.8222\;\times \;10^{-5}\right)$	$\left(1.5197\;\times \;10^{-5}\right)$	$\left(2.4786\;\times \;10^{-4}\right)$	$\left(5.1903\;\times \;10^{-5}\right)$	$\left(1.5991\;\times \;10^{-5}\right)$
$k_{2}$ (mm)	0	1.3808$\;\times \;10^{-6}$	5.2682$\;\times \;10^{-8}$	4.4839$\;\times \;10^{-8}$	2.5099$\;\times \;10^{-6}$	5.4053$\;\times \;10^{-8}$	2.9584$\;\times \;10^{-8}$
		$\left(7.9395\;\times \;10^{-6}\right)$	$\left(1.5151\;\times \;10^{-6}\right)$	$\left(4.4062\;\times \;10^{-7}\right)$	$\left(9.5927\;\times \;10^{-6}\right)$	$\left(1.2615\;\times \;10^{-6}\right)$	$\left(4.2352\;\times \;10^{-7}\right)$
Residual (mm)	0	1.1286	0.1127	1.1434$\;\times \;10^{-2}$	1.1246	0.1140	1.1523$\;\times \;10^{-2}$
		(0.1116)	$\left(1.0592\;\times \;10^{-2}\right)$	$\left(1.2037\;\times \;10^{-3}\right)$	(0.10750)	$\left(1.1194\;\times \;10^{-2}\right)$	$\left(1.1441\;\times \;10^{-3}\right)$
Iterations	-	32.66	35.62	19.06	52.97	47.62	28.38
		(16.08)	(12.04)	(3.8778)	(19.37)	(6.93)	(9.04)

**Table 4:** Evaluation of the calibration method with noise.

Lens 2		With Corrections			Without Corrections		
SNR (dB)	Ideal	30	40	50	30	40	50
$C_{x}$ (mm)	0.5	0.5248	0.5015	0.5001	0.5173	0.4984	0.4995
		(9.9308$\;\times \;10^{-2}$)	(2.7468$\;\times \;10^{-2}$)	(9.7033$\;\times \;10^{-3}$)	(0.1247)	$\left(3.6729\;\times \;10^{-2}\right)$	$\left(1.1802\;\times \;10^{-2}\right)$
$C_{y}$ (mm)	−0.7	−0.6579	−0.6975	−0.6977	−0.6431	−0.6896	−0.6984
		(0.1297)	$\left(3.6233\;\times \;10^{-2}\right)$	$\left(1.0635\;\times \;10^{-2}\right)$	(0.1238)	$\left(3.9594\;\times \;10^{-2}\right)$	$\left(1.1298\;\times \;10^{-2}\right)$
*f* (mm)	8	8.1090	8.0213	7.9961	8.0267	7.9991	7.9968
		(0.3833)	(0.1061)	$\left(3.9105\;\times \;10^{-2}\right)$	(0.4084)	(0.1297)	$\left(4.0700\;\times \;10^{-2}\right)$
$k_{1}$ (mm)	1$\;\times \;10^{-2}$	9.9109$\;\times \;10^{-3}$	9.9923$\;\times \;10^{-3}$	9.9992$\;\times \;10^{-3}$	0.9873$\;\times \;10^{-3}$	0.9987$\;\times \;10^{-3}$	9.9982$\;\times \;10^{-3}$
		$\left(3.4371\;\times \;10^{-4}\right)$	$\left(1.0460\;\times \;10^{-4}\right)$	$\left(3.5837\;\times \;10^{-5}\right)$	$\left(3.6840\;\times \;10^{-4}\right)$	$\left(1.0071\;\times \;10^{-4}\right)$	$\left(3.1988\;\times \;10^{-5}\right)$
$k_{2}$ (mm)	3$\;\times \;10^{-5}$	2.4701$\;\times \;10^{-5}$	2.4541$\;\times \;10^{-4}$	2.9964$\;\times \;10^{-5}$	2.3909$\;\times \;10^{-5}$	2.9938$\;\times \;10^{-5}$	3.0155$\;\times \;10^{-5}$
		$\left(1.4913\;\times \;10^{-5}\right)$	$\left(4.2144\;\times \;10^{-6}\right)$	$\left(1.4822\;\times \;10^{-6}\right)$	$\left(1.4521\;\times \;10^{-5}\right)$	$\left(4.8994\;\times \;10^{-6}\right)$	$\left(1.4209\;\times \;10^{-6}\right)$
Residual (mm)	0	1.3890	0.1398	1.3961 $\;\times \;10^{-2}$	1.4080	0.1404	1.4182 $\;\times \;10^{-2}$
		(0.1491)	$\left(1.3390\;\times \;10^{-2}\right)$	$\left(1.3622\;\times \;10^{-3}\right)$	(0.1361)	$\left(1.4854\;\times \;10^{-2}\right)$	$\left(1.3964\;\times \;10^{-5}\right)$
Iterations	-	23.44	15.72	11.64	41.72	40.98	42.40
		(16.08)	(12.04)	(3.8778)	(19.37)	(6.93)	(9.04)

**Table 5:** Evaluation of the calibration method with imbalances and noise.

Imbalance	Lens 1			
	Ideal	40 dB	50 dB	Without Noise
$C_{x}$ (mm)	0.5	0.5463	0.4859	0.5284
		(1.1246)	(1.1184)	(1.0543)
$C_{y}$ (mm)	−0.7	−0.7627	−0.7442	−0.6862
		(1.2792)	(1.1955)	(1.0025)
*f* (mm)	8	24.5821	24.7798	24.7831
		(0.4489)	(0.3366)	(0.2916)
$k_{1}$ (mm)	2$\;\times \;10^{-3}$	2.008$\;\times \;10^{-3}$	1.9911$\;\times \;10^{-3}$	1.9964$\;\times \;10^{-3}$
		$\left(5.5946\;\times \;10^{-5}\right)$	$\left(4.9795\;\times \;10^{-5}\right)$	$\left(3.0805\;\times \;10^{-5}\right)$
$k_{2}$ (mm)	0	6.3620$\;\times \;10^{-7}$	8.9598$\;\times \;10^{-8}$	3.3083$\;\times \;10^{-8}$
		$\left(1.7950\;\times \;10^{-6}\right)$	$\left(1.2040\;\times \;10^{-6}\right)$	$\left(1.1553\;\times \;10^{-6}\right)$
Residual (mm)	0	6.1689$\;\times \;10^{-2}$	5.1177$\;\times \;10^{-2}$	4.8745$\;\times \;10^{-2}$
		$\left(1.2298\;\times \;10^{-2}\right)$	$\left(5.1542\;\times \;10^{-3}\right)$	$\left(3.9578\;\times \;10^{-3}\right)$
iterations	-	35.72	24.19	24.72
		(9.85)	(10.86)	(10.40)

**Table 6:** Evaluation of the calibration method with imbalances and noise.

Imbalance	Lens 2			
	Ideal	40 dB	50 dB	Without Noise
$C_{x}$ (mm)	0.5	0.5383	0.4881	0.4972
		(0.3093)	(0.3005)	(0.2963)
$C_{y}$ (mm)	−0.7	−0.7078	−0.6543	−0.6723
		(0.3339)	(0.2862)	(0.2774)
*f* (mm)	8	7.9301	7.9546	7.9816
		(0.1746)	(0.1036)	(0.1066)
$k_{1}$ (mm)	1$\;\times \;10^{-2}$	0.9950$\;\times \;10^{-2}$	1.012$\;\times \;10^{-3}$	0.9997$\;\times \;10^{-2}$
		$\left(1.4376\;\times \;10^{-4}\right)$	$\left(1.3249\;\times \;10^{-4}\right)$	$\left(1.2099\;\times \;10^{-4}\right)$
$k_{2}$ (mm)	3$\;\times \;10^{-5}$	2.9671$\;\times \;10^{-5}$	2.9786$\;\times \;10^{-5}$	2.8683$\;\times \;10^{-5}$
		$\left(5.7399\;\times \;10^{-6}\right)$	$\left(5.0512\;\times \;10^{-6}\right)$	$\left(4.7542\;\times \;10^{-6}\right)$
Residual (mm)	0	4.6661$\;\times \;10^{-2}$	3.9131$\;\times \;10^{-2}$	2.3292$\;\times \;10^{-2}$
		$\left(3.2799\;\times \;10^{-3}\right)$	$\left(2.5302\;\times \;10^{-3}\right)$	$\left(2.7743\;\times \;10^{-3}\right)$
Iterations	-	17.08	12.87	10.97
		(0.1710)	(7.5334)	(3.20)

**Table 7:** Evaluation of the calibration method with PSD distortion, imbalances and noise.

	Ideal	PSD Distortion	PSD Distortion and SNR 40 dB	PSD Distortion and Imbalances	PSD Distortion, Imbalances and SNR 40 dB
$C_{x}$ (mm)	0.5	0.5016	0.4812	0.4105	0.3840
			(0.1564)	(1.4487)	(1.5763)
$C_{y}$ (mm)	−0.7	−0.7034	−0.6817	−0.7933	−0.8345
			(0.1216)	(1.4161)	(1.5071)
*f* (mm)	25	25.0412	25.0543	24.8257	24.8345
			(0.1403)	(0.1965)	(0.2575)
$k_{1}$ (mm)	6.9416$\;\times \;10^{-4}$	6.9525$\;\times \;10^{-4}$	6.8486$\;\times \;10^{-4}$	6.9778$\;\times \;10^{-4}$	6.9800$\;\times \;10^{-4}$
			$\left(2.9148\;\times \;10^{-5}\right)$	$\left(1.5191\;\times \;10^{-5}\right)$	$\left(3.2099\;\times \;10^{-5}\right)$
$k_{2}$ (mm)	0	6.6503$\;\times \;10^{-9}$	2.7347$\;\times \;10^{-7}$	9.0506$\;\times \;10^{-8}$	9.9325$\;\times \;10^{-8}$
			$\left(8.0904\;\times \;10^{-7}\right)$	$\left(2.4403\;\times \;10^{-7}\right)$	$\left(5.9951\;\times \;10^{-7}\right)$
Residual (mm)	0	2.4690$\;\times \;10^{-6}$	1.0088$\;\times \;10^{-2}$	2.5190$\;\times \;10^{-3}$	0.1026
			$\left(9.9455\;\times \;10^{-3}\right)$	$\left(3.0651\;\times \;10^{-3}\right)$	$\left(1.1644\;\times \;10^{-2}\right)$
Iterations	-	15	31.80	34.52	35.14
			(10.29)	(13.60)	(15.4214)

**Table 8:** Evaluation of the calibration method with PSD distortion, imbalances and noise.

	Ideal	PSD Distortion	PSD Distortion and SNR 40 dB	PSD Distortion and Imbalances	PSD Distortion, Imbalances and SNR 40 dB
$C_{x}$ (mm)	0.5	0.5039	0.5013	0.4842	0.5048
			(3.1690$\;\times \;10^{-2}$)	(0.3306)	(0.3733)
$C_{y}$ (mm)	−0.7	−0.6993	−0.6926	−0.7254	−0.6807
			(4.3307$\;\times \;10^{-2}$)	(0.3232)	(0.3158)
*f* (mm)	25	8.0146	8.0175	7.9482	8.1176
			(0.1441)	(0.2061)	(0.3547)
$k_{1}$ (mm)	8.6941$\;\times \;10^{-3}$	8.7203$\;\times \;10^{-3}$	8.7065$\;\times \;10^{-3}$	8.7226$\;\times \;10^{-3}$	8.5761$\;\times \;10^{-3}$
			$\left(1.0378\;\times \;10^{-4}\right)$	$\left(1.2086\;\times \;10^{-4}\right)$	$\left(8.6034\;\times \;10^{-4}\right)$
$k_{2}$ (mm)	−3$\;\times \;10^{-5}$	2.9893$\;\times \;10^{-5}$	2.9708$\;\times \;10^{-5}$	2.8592$\;\times \;10^{-5}$	2.8139$\;\times \;10^{-5}$
			$\left(4.0140\;\times \;10^{-6}\right)$	$\left(4.5308\;\times \;10^{-6}\right)$	$\left(6.5081\;\times \;10^{-6}\right)$
Residual (mm)	0	5.8242$\;\times \;10^{-5}$	1.2502$\;\times \;10^{-2}$	1.6771$\;\times \;10^{-2}$	3.5957$\;\times \;10^{-4}$
			$\left(1.2933\;\times \;10^{-2}\right)$	$\left(1.8478\;\times \;10^{-2}\right)$	$\left(1.8663\;\times \;10^{-3}\right)$
Iterations	-	10	13.81	11.44	16.19
			(4.85)	(1.53)	(8.62)

**Table 9:** Comparison results.

	Ideal	SNR 40 dB	SNR 40 dB and Imbalances	SNR 40 dB and PSD Distortion	SNR 40 dB, Imbalances and PSD Distortion	SNR 50 dB and PSD Distortion (Practical Case)
$C_{x}$ (mm)	0.5	0.5062	0.5463	0.4812	0.3840	0.5060
		(0.1551)	(1.1246)	(0.1564)	(1.5763)	$\left(4.3929\;\times \;10^{-2}\right)$
$C_{y}$ (mm)	−0.7	−0.6806	−0.7627	−0.6817	−0.8345	−0.6920
		(0.2219)	(1.2792)	(0.1216)	(1.5071)	$\left(7.2875\;\times \;10^{-2}\right)$
*f* (mm)	8	24.9868	24.5821	25.0543	24.8345	24.9954
		(0.1724)	(0.4489)	(0.1403)	(0.2575)	$\left(5.4405\;\times \;10^{-3}\right)$
$k_{1}$ (mm)	*	1.9962$\;\times \;10^{-3}$	−2.008$\;\times \;10^{-3}$	6.8486$\;\times \;10^{-4}$	6.9800$\;\times \;10^{-4}$	6.93531$\;\times \;10^{-4}$
		$\left(4.8222\;\times \;10^{-5}\right)$	$\left(5.5946\;\times \;10^{-5}\right)$	$\left(2.9148\;\times \;10^{-5}\right)$	$\left(3.2099\;\times \;10^{-5}\right)$	$\left(9.0483\;\times \;10^{-6}\right)$
$k_{2}$ (mm)	0	5.2682$\;\times \;10^{-8}$	6.3620$\;\times \;10^{-7}$	2.7347$\;\times \;10^{-7}$ *	9.9325$\;\times \;10^{-8}$ *	3.8829$\;\times \;10^{-8}$
		$\left(1.5151\;\times \;10^{-6}\right)$	$\left(1.7950\;\times \;10^{-6}\right)$	$\left(8.0904\;\times \;10^{-7}\right)$	$\left(5.9951\;\times \;10^{-7}\right)$	$\left(2.5433\;\times \;10^{-7}\right)$
Residual (mm)	0	0.1127	6.1689$\;\times \;10^{-2}$	1.0088$\;\times \;10^{-4}$	0.1026	1.0000$\;\times \;10^{-2}$
		$\left(1.0592\;\times \;10^{-2}\right)$	$\left(1.2298\;\times \;10^{-2}\right)$	$\left(9.9455\;\times \;10^{-3}\right)$	$\left(1.0604\;\times \;10^{-2}\right)$	$\left(1.2037\;\times \;10^{-3}\right)$
Iterations	-	35.62	35.72	31.80	35.14	15.05
		(12.04)	(9.85)	(10.29)	(15.4214)	(0.9252)

* The ideal value of the $k_{1}$ parameter is 2$\;\times \;10^{-3}$ for the first two columns and 6.9416$\;\times \;10^{-4}$ for the last three columns.

**Table 10:** Comparison results.

	Ideal	SNR 40 dB	SNR 40 dB and Imbalances	SNR 40 dB and PSD Distortion	SNR 40 dB, Imbalances and PSD Distortion	SNR 50 dB and PSD Distortion (Practical Case)
$C_{x}$ (mm)	0.5	0.5015	0.5383	0.5013	0.5048	0.5001
		(2.7468$\;\times \;10^{-2}$)	(0.3093)	(3.1690$\;\times \;10^{-2}$)	(0.3733)	(9.7033$\;\times \;10^{-3}$)
$C_{y}$ (mm)	−0.7	−0.6975	−0.7078	−0.6926	−0.6807	−0.6977
		$\left(3.6233\;\times \;10^{-2}\right)$	(0.3339)	$\left(4.3307\;\times \;10^{-2}\right)$	(0.3158)	$\left(1.0635\;\times \;10^{-2}\right)$
*f* (mm)	8	8.0213	7.9301	8.0175	8.1176	7.9961
		(0.1061)	(0.1746)	(0.1441)	(0.3547)	$\left(3.9105\;\times \;10^{-2}\right)$
$k_{1}$ (mm)	*	9.9923$\;\times \;10^{-3}$	9.9503$\;\times \;10^{-3}$	8.7065$\;\times \;10^{-3}$	8.5761$\;\times \;10^{-3}$	8.7192$\;\times \;10^{-2}$
		$\left(1.0460\;\times \;10^{-4}\right)$	$\left(1.4376\;\times \;10^{-4}\right)$	$\left(1.0378\;\times \;10^{-4}\right)$	$\left(8.6034\;\times \;10^{-4}\right)$	$\left(3.1025\;\times \;10^{-5}\right)$
$k_{2}$ (mm)	3$\;\times \;10^{-5}$	2.4541$\;\times \;10^{-5}$	2.9671$\;\times \;10^{-5}$	2.9708$\;\times \;10^{-5}$	2.8139$\;\times \;10^{-5}$	3.0093$\;\times \;10^{-2}$
		$\left(4.2144\;\times \;10^{-6}\right)$	$\left(5.7399\;\times \;10^{-6}\right)$	$\left(4.0140\;\times \;10^{-6}\right)$	$\left(6.5081\;\times \;10^{-6}\right)$	$\left(1.1896\;\times \;10^{-6}\right)$
Residual (mm)	0	0.1398	4.6661$\;\times \;10^{-2}$	1.2502$\;\times \;10^{-2}$	3.5957$\;\times \;10^{-4}$	1.3961$\;\times \;10^{-2}$
		$\left(1.3390\;\times \;10^{-2}\right)$	$\left(3.2799\;\times \;10^{-3}\right)$	$\left(1.2933\;\times \;10^{-2}\right)$	$\left(1.8663\;\times \;10^{-3}\right)$	$\left(1.3622\;\times \;10^{-3}\right)$
Iterations	-	15.72	17.08	13.81	16.19	10.60
		(2.75)	(7.5334)	(4.85)	(8.62)	(0.695)

* The ideal value of the $k_{1}$ parameter is 1$\;\times \;10^{-2}$ for the first two columns and 8.6941$\;\times \;10^{-3}$ for the last three columns.

**Table 11:** Results for Lens 1.

Number of Images	8	9	10	11	12
Residuals (mm)	0.1014	0.2244	0.1969	0.2398	0.2844
$C_{x}$ (mm)	−0.1617	−1.1492	−0.0046	−0.0401	−0.0289
$C_{y}$ (mm)	−0.2377	0.8442	−0.0903	−0.1230	−0.1471
*f* (mm)	40.5751	39.4077	39.1392	39.1994	39.1892
*γ*	−0.1827	−0.1153	−0.1304	−0.1366	−0.1350
$K_{1}$ (mm)	−1.0499$\;\times \;10^{-3}$	8.1831$\;\times \;10^{-5}$	−7.1116$\;\times \;10^{-4}$	−7.0715$\;\times \;10^{-4}$	−7.1402$\;\times \;10^{-4}$
$K_{2}$ (mm)	9.3220$\;\times \;10^{-5}$	−2.0399$\;\times \;10^{-5}$	4.4269$\;\times \;10^{-5}$	4.1609$\;\times \;10^{-5}$	1.7593$\;\times \;10^{-5}$
$P_{1}$ (mm)	−5.9407$\;\times \;10^{-5}$	3.6661$\;\times \;10^{-5}$	−5.6560$\;\times \;10^{-5}$	−8.0734$\;\times \;10^{-5}$	−1.0785$\;\times \;10^{-4}$
$P_{2}$ (mm)	−3.1457$\;\times \;10^{-4}$	−2.7414$\;\times \;10^{-4}$	−2.4884$\;\times \;10^{-4}$	−2.5716$\;\times \;10^{-4}$	−2.1089$\;\times \;10^{-4}$
Iterations	18	15	16	19	28

**Table 12:** Results Lens 2.

Number of Images	8	9	10	11	12
Residuals (mm)	1.4599$\;\times \;10^{-3}$	0.1227	4.8331$\;\times \;10^{-2}$	0.1899	0.2175
$C_{x}$ (mm)	−0.1471	−0.1999	−0.2211	−0.1703	−0.1799
$C_{y}$ (mm)	0.1586	0.1536	−0.0508	−0.1085	−0.1785
*f* (mm)	8.1318	7.8380	7.0705	7.5267	7.4933
*γ*	−5.4091$\;\times \;10^{-3}$	−3.6463$\;\times \;10^{-3}$	−8.2606$\;\times \;10^{-3}$	1.1067$\;\times \;10^{-4}$	1.3078$\;\times \;10^{-4}$
$K_{1}$ (mm)	−2.7808$\;\times \;10^{-3}$	−2.6163$\;\times \;10^{-3}$	−2.3022$\;\times \;10^{-3}$	−1.6412$\;\times \;10^{-4}$	−1.5478$\;\times \;10^{-3}$
$K_{2}$ (mm)	−1.0672$\;\times \;10^{-4}$	−1.1567$\;\times \;10^{-4}$	−1.6086$\;\times \;10^{-4}$	−2.1803$\;\times \;10^{-4}$	−2.2796$\;\times \;10^{-4}$
$K_{3}$ (mm)	1.3404$\;\times \;10^{-6}$	2.1313$\;\times \;10^{-6}$	4.2200$\;\times \;10^{-6}$	5.8473$\;\times \;10^{-6}$	6.3565$\;\times \;10^{-6}$
$P_{1}$ (mm)	1.4247$\;\times \;10^{-4}$	2.9718$\;\times \;10^{-4}$	2.3381$\;\times \;10^{-4}$	2.2626$\;\times \;10^{-4}$	2.2400$\;\times \;10^{-4}$
$P_{2}$	−4.4236$\;\times \;10^{-4}$	−1.1882$\;\times \;10^{-4}$	5.5282$\;\times \;10^{-4}$	3.9177$\;\times \;10^{-4}$	3.2860$\;\times \;10^{-4}$
Iterations	58	35	30	22	23
