# Erratum: Rodríguez-Navarro, D., et al. Mathematical Model and Calibration Procedure of a PSD Sensor Used in Local Positioning Systems. *Sensors* 2016, *16*, 1484

**DOI:** 10.3390/s16111788

**Published:** 2016-10-26

**Authors:** David Rodríguez-Navarro, José Luis Lázaro-Galilea, Ignacio Bravo-Muñoz, Alfredo Gardel-Vicente, Francisco Domingo-Perez, Georgios Tsirigotis

**Affiliations:** 1 Department of electronics, University of Alcalá, Alcalá de Henares, Madrid 28801, Spain; Lazaro@depeca.uah.es (J.L.L.-G.); ibravo@depeca.uah.es (I.B.-M.); alfredo@depeca.uah.es (A.G.-V.); fco.domingo@depeca.uah.es (F.D.-P.); 2 Informatics Engineering Department, Eastern Macedonia and Thrace Institute of Technology, Kvala 65404, Greece; tsirigo@teikav.edu.gr

The authors wish to make the following correction to their paper [1]: Reference 2 should read: Braun, J.; Štroner, M.; Urban, R.; Dvořáček, F. Suppression of systematic errors of electronic distance meters for measurement of short distances. *Sensors* **2015**, *15*, 19264–19301.

The manuscript will be updated and the original will remain online on the article webpage. The authors would like to apologize for any inconvenience caused. 
